# Exploring barriers to colorectal cancer screening in Saudi Arabia: findings from a cross-sectional study

**DOI:** 10.3389/fpubh.2025.1601592

**Published:** 2025-07-25

**Authors:** Saleh Busbait

**Affiliations:** Department of Surgery, Imam Abdulrahman Bin Faisal University, Dammam, Saudi Arabia

**Keywords:** colorectal cancer, screening barriers, awareness, psychological factors, public health

## Abstract

**Background:**

Colorectal cancer (CRC) is a leading cause of cancer-related morbidity and mortality worldwide. Despite national screening recommendations, CRC screening uptake remains low in Saudi Arabia. This study aims to identify perceived barriers to CRC screening and examine their demographic variations.

**Methods:**

A cross-sectional study was conducted with 412 adults in the Eastern Province of Saudi Arabia. The study was conducted between April 2024 and July 2024 using a self-administered questionnaire. Perceived barriers to CRC were assessed using a questionnaire adapted from prior published studies. Statistical analyses included chi-square tests, exploratory factor analysis (EFA), and logistic regression to determine demographic predictors of screening barriers.

**Results:**

The most frequently reported barriers clustered into three domains: Personal Fears, Lack of Knowledge, and Healthcare System Barriers. “Absence of symptoms” (61.9%) and “fear of results” (28.9%) loaded under Personal Fears; “lack of awareness” (39.1%) under Lack of Knowledge; and “insufficient public awareness campaigns” (35.7%) under Healthcare Barriers. Women more commonly reported fear-related concerns, while younger participants cited knowledge gaps and financial limitations. The three factors explained 77.6% of the total variance. Logistic regression indicated that younger age and lack of prior screening experience were significant predictors of higher perceived barriers (*p* < 0.05).

**Conclusion:**

The findings highlight the need for targeted interventions addressing psychological concerns, increasing public awareness, and improving healthcare provider engagement. Addressing these barriers through structured awareness campaigns, provider-driven screening initiatives, and improved access to non-invasive screening options could increase CRC screening rates and early detection in Saudi Arabia.

## 1 Introduction

Colorectal cancer (CRC) ranks among the most prevalent malignancies worldwide and second leading cause of cancer related death globally (1). In Saudi Arabia, it is most commonly diagnosed around the ages of 55 and 60 (1). Screening for CRC has the potential to decrease mortality by 50% (2). Although the national screening program recommends beginning CRC screening at age 45, uptake and adherence is low, which poses a significant public health problem (3). Previous research has indicated that lack of knowledge of the risk factors, screening methods, and preventive measures of CRC are some of the main reasons for the low rates of screening (1). Psychological barriers such as fear of the outcome, pain, and shame affect women most (4). Moreover, lack of physician recommendations, absence of symptoms, financial constraints, and limited access to screening facilities contribute to the ongoing low rates of screening (3).

Colorectal cancer (CRC) poses a growing public health challenge in Saudi Arabia, with the age-standardized incidence rate increasing from approximately 5 to 8.5 cases per 100,000 person-years between the late 1990s and 2017, indicating a rising burden over the past two decades (5, 6). Notably, about 28% of CRC cases occur in patients younger than 50 years, underscoring the importance of including younger populations in screening and awareness efforts due to the increasing incidence of early-onset CRC (5, 7). The median age at diagnosis is 58 years, with most patients being married and predominantly residing in the Riyadh, Makkah, and Eastern provinces—regions recognized as CRC hotspots within the country (6). Alarmingly, 30% of patients present with advanced-stage disease, highlighting gaps in early detection and screening practices (7).

While several studies in Saudi Arabia have investigated barriers to colorectal cancer screening, most have focused on descriptive analyses with limited demographic comparisons (4, 8–11). Notably, only one other study has employed factor analysis to comprehensively categorize these barriers. Our study expands on this by applying factor analysis alongside demographic stratification, providing deeper insights to guide tailored interventions.

Understanding the barriers to CRC screening is essential for developing targeted interventions aimed at improving screening adherence and reducing CRC-related mortality. Even though CRC awareness has been assessed in Saudi Arabia, few studies have evaluated the barriers to screening and demographic correlates (2). To help fill this gap, this study aims to explore the main barriers to CRC screening and their relationship with demographic variables, such as gender, age, and educational level.

## 2 Materials and methods

This cross-sectional study was conducted to determine the perceived barriers to CRC screening among the residents of the Eastern Province of Saudi Arabia between April 2024 and July 2024. A self-administered electronic questionnaire was delivered through social media platforms and physically in public places.

To be eligible for the study, the participants had to be 18 years or older, living in the Eastern Province, and have no previous history of CRC or IBD. Although national guidelines recommend CRC screening starting at age 45, participants aged 18 and older were included to capture barriers among younger adults, reflecting the increasing incidence of early-onset CRC. This approach helps explore emerging obstacles in an at-risk group likely to be targeted by future screening efforts. Exclusion criteria included participants were illiterate or unable to complete the questionnaire or refused to participate. The minimum required sample size was calculated using the Raosoft sample size calculator with a 5% margin of error and 95% confidence interval, which gave a calculated sample size of 385 participants.

The questionnaire was developed from earlier published tools including international and regional studies on CRC screening barriers. It was divided into three main sections: socio-demographic data, CRC awareness and screening history, and perceived barriers to CRC screening. Age, gender, area of residence, and educational level were collected in the socio-demographic section. The provinces were further divided into core (urban) and peripheral regions based on the provincial administrative structure. Participants’ prior knowledge of CRC, family history, screening history, and knowledge of recommended screening were assessed in the CRC awareness and screening history section. The barriers section assessed 15 possible barriers to CRC screening across three domains: psychological, knowledge-based, and healthcare system barriers.

The perceived barrier items in our questionnaire were adapted from Galal et al. (9), who themselves drew on constructs from Berkowitz et al. (12) and Hoffman et al. (13). Galal et al. (9) conducted an Exploratory Factor Analysis (EFA) and grouped the items into domains based on the factor structure observed in their population.

While we retained most of their items, we made several wording modifications to enhance clarity and cultural relevance for our target population. For example, “lack of CRC symptoms” was adapted from “absence of signs and symptoms,” “busy” replaced “lack of time,” and “lack of confidence in physicians” was rephrased from “healthcare providers are not trustworthy.” These adjustments were intended to improve comprehension without altering the underlying barrier constructs.

Importantly, we conducted our own independent EFA using our dataset, rather than applying Galal’s domain structure. Our three domains—Personal Fears, Lack of Knowledge, and Healthcare System Barriers—were empirically derived and reflect population-specific factor patterns, as expected in EFA-based analysis. Additionally, we employed a binary Yes/No response format to reduce cognitive burden, whereas they used a three-option format (Yes/No/Not Sure). The original questionnaire was pilot tested to ensure clarity and reliability. The online survey format facilitated and prevented incomplete submissions, resulting in no missing data.

The clarity and reliability of the questionnaire was tested using a pilot study with 14 randomly selected participants. The internal consistency of the barriers scale was determined by Cronbach’s alpha, which was 0.74, indicating acceptable reliability.

Participants were recruited only after approval of the study by the institutional review board. Participants gave their informed consent before completing the survey, which was done anonymously and confidentially. There was no physical, psychological, social, legal, or economic risk to participants involved in the study.

IBM SPSS version 30.0 was used for data analysis. Descriptive statistics were used to present participant characteristics and perceived barriers. Chi-square tests were used to determine the relationship between the independent variables (gender, age, and education) and the dependent variables (perceived barriers). Exploratory factor analysis (EFA) was conducted using principal component analysis (PCA) with varimax rotation to identify underlying barrier domains. This approach was selected as it is appropriate for binary (yes/no) questionnaire items, facilitating the reduction of variables into interpretable factors while accommodating the categorical nature of the data. This exploratory, statistical approach does not rely on a predefined theoretical behavioral model but is designed to empirically discover and categorize barrier groups to better inform tailored intervention strategies Logistic regression was used to determine the predictors of high barriers, including demographics and screening history in the model. The total barrier score was dichotomized into ‘high’ and ‘low’ categories using the median value of 3 as the cutoff, ensuring balanced groups for logistic regression analysis.

## 3 Results

Among 412 participants, 70.6% were male. The largest age groups were 41–50 years (26.2%) and 31–40 years (24.5%), while 15.0% were aged 18–30. Regarding educational attainment, most participants had a undergraduate degree (66.0%), while 17.7% had completed secondary school, and 14.6% had attained postgraduate degree. Only a small proportion had only intermediate (1.0%) or primary education (0.7%) (Table 1). Although 20.9% of participants reported having a family history of CRC, only 11.7% had undergone CRC screening, highlighting the low uptake of screening among the study population.

**Table 1:** Socio-demographic characteristics and screening uptake.

Socio-demographic and CRC screening variables	Number	%
Age		
18–30	62	15.0
31–40	101	24.5
41–50	108	26.2
51–60	88	21.4
>60	53	12.9
Total	412	100
Gender		
Male	291	70.6
Female	121	29.4
Total	412	100
Residence		
Core (main)	387	93.9
Peripheral	25	6.1
Total	412	100
Education level		
Primary	3	0.7
Intermediate	4	1.0
Secondary	73	17.7
Undergraduate degree	272	66.0
Postgraduate degree	60	14.6
Family history of CRC?	86	20.9
Ever screened for CRC?	48	11.7

The most common barriers to colorectal cancer (CRC) screening were categorized into three key domains. In the Personal Fears domain, notable barriers included fear of results (28.9%), fear of painful procedures (20.6%), and shyness (26.5%). The Lack of Knowledge domain was characterized by absence of symptoms (61.9%), lack of CRC awareness (39.1%), and insufficient public health campaigns (35.7%). The Healthcare Barriers domain, though less frequently reported, included lack of screening facilities (24.0%), financial concerns (16.3%), and transportation difficulties (5.6%) (Table 2).

**Table 2:** Perceived barriers to colorectal cancer screening.

List of barrier to CRC screening	Frequency	Percentage (%)
Fear of results	119	28.9
Fear of painful procedures	85	20.6
Shyness	109	26.5
Lack of time	105	25.5
Previous bad experience with screening	16	3.9
Absence of signs and symptoms of CRC	255	61.9
Lack of awareness about symptoms and signs	161	39.1
Feeling unrest in dealing with doctors	40	9.7
Lack of knowledge about the tests	118	28.6
Lack of specialized facilities	99	24.0
Lack of transportation	23	5.6
Lack of public awareness of CRC screening	147	35.7
Lack of providers’ knowledge about recommended screening	41	10.0
Health care providers are not trustworthy	33	8.0
Financial burden and cost of screening	67	16.3

Chi-square tests were conducted to assess the associations between demographic factors (gender, age, and education) and perceived barriers to CRC screening. Table 3 presents the *p*-values for each barrier across these variables. Significant gender differences were observed in several barriers. Fear-related barriers within the Personal Fears domain were significantly more prevalent among women than men. Specifically, women reported a higher frequency of fear of results (32.5% vs. 24.8%, *p* = 0.021), fear of painful procedures (27.9% vs. 19.1%, *p* = 0.034), and shyness or embarrassment (31.8% vs. 22.4%, *p* = 0.012) compared to their male counterparts. Additionally, previous bad experience with screening was more commonly cited by women (5.1%) than by men (2.9%) (*p* = 0.049).

**Table 3:** Chi-square analysis of barriers by gender, age, and education.

List of barrier to CRC screening	Gender (*p*-value)	Age (*p*-value)	Education (*p*-value)
Fear of results	0.021*	0.087	0.209
Fear of painful procedures	0.034*	0.112	0.173
Shyness	0.012*	0.265	0.381
Lack of time	0.001*	0.152	0.320
Previous bad experience with screening	0.049*	0.219	0.442
Absence of signs and symptoms of CRC	0.092	0.011*	0.331
Lack of awareness about symptoms and signs	0.118	0.018*	0.235
Feeling unrest in dealing with doctors	0.101	0.045*	0.193
Lack of knowledge about the tests	0.086	0.078	<0.001*
Lack of specialized facilities	0.156	0.097	0.022*
Lack of transportation	0.207	0.156	0.312
Lack of public awareness of CRC screening	0.204	0.127	0.028*
Lack of providers’ knowledge about recommended screening	0.311	0.148	0.041*
Health care providers are not trustworthy	0.102	0.222	0.132
Financial burden and cost of screening	0.072	0.037*	0.267

*p < 0.05 indicates statistical significance (marked with *).

Age was significantly associated with more than one screening barrier. Lack of awareness about CRC symptoms and signs was more frequently reported by younger participants (≤40 years: 46.2%) than by older participants (>40 years: 32.7%) (*p* = 0.018). Similarly, feeling unrest in dealing with doctors was more prevalent among younger individuals (≤40 years: 14.1% vs. >40 years: 6.3%, *p* = 0.045). Financial burden and cost of screening was also significantly associated with younger age groups (≤40 years: 21.5% vs. >40 years: 10.7%, *p* = 0.037). Finally, absence of symptoms as a reason to delay screening was a more frequent barrier in younger individuals (≤40 years: 15.8% vs. >40 years: 7.1%, *p* = 0.011), suggesting that financial constraints and lack of perceived need for screening are key deterrents in this population.

Lower educational attainment was significantly associated with increased barriers across both the Lack of Knowledge and Healthcare Barriers domains. Participants with primary or secondary education were more likely to report lack of knowledge about screening tests compared to those with undergraduate or postgraduate degrees (48.9% vs. 22.4%, *p* < 0.001). Similarly, they more frequently cited healthcare-related obstacles such as lack of specialized facilities (31.3% vs. 18.2%, *p* = 0.022), limited public awareness about CRC screening (42.6% vs. 28.7%, *p* = 0.028), and insufficient healthcare provider knowledge regarding CRC screening (19.7% vs. 10.4%, *p* = 0.041).

A principal component analysis (PCA) with Varimax rotation was conducted to explore the underlying structure of perceived barriers to CRC screening. Based on eigenvalues greater than 1, three distinct factors were extracted, collectively explaining 77.6% of the total variance (Factor 1: 34.8%, Factor 2: 23.7%, Factor 3: 19.1%). The Kaiser–Meyer–Olkin measure of sampling adequacy was 0.678, and Bartlett’s test of sphericity was significant (*p* < 0.001), confirming that the dataset was suitable for factor analysis (Table 4).

**Table 4:** Factor analysis of perceived barriers to CRC screening.

Perceived barriers	Factor 1: personal fears	Factor 2: lack of knowledge	Factor 3: healthcare barriers	Communality
Fear of results	0.831			0.636
Shyness	0.830			0.477
Fear of painful procedures	0.772			0.612
Lack of confidence in physicians			0.468	0.567
Lack of health education and awareness		0.807		0.577
Lack of providers’ knowledge about screening		0.726		0.380
Absence of signs and symptoms of CRC		0.560		0.730
Previous bad experience with screening			0.720	0.413
Financial burden and cost of screening			0.651	0.396
Lack of time (Busy)			0.532	0.485
Eigenvalue	2.951	2.132	1.616	
Cronbach’s Alpha	0.764	0.611	0.522	
% Variance Explained	34.8%	23.7%	19.1%	

Only barriers with factor loadings ≥ 0.4 are included.

The first factor, Personal Fears, accounted for the largest proportion of variance (34.8%) and had the highest Cronbach’s alpha (0.764), reflecting good internal consistency. This factor included fear of results (loading = 0.831, communality = 0.636), shyness (loading = 0.830, communality = 0.477), and fear of painful procedures (loading = 0.772, communality = 0.612). The second factor, Lack of Knowledge, explained 23.7% of the variance and had a Cronbach’s alpha of 0.611, indicating moderate reliability. It included lack of health education and awareness (loading = 0.807, communality = 0.577), lack of providers’ knowledge about screening (loading = 0.726, communality = 0.380), and absence of signs and symptoms of CRC (loading = 0.560, communality = 0.730). The third factor, Healthcare Barriers, contributed 19.1% of the variance but had the lowest Cronbach’s alpha (0.522). This factor included previous bad experiences with screening (loading = 0.720, communality = 0.413), financial burden and cost of screening (loading = 0.651, communality = 0.396), and lack of time (loading = 0.532, communality = 0.485).

A binary logistic regression analysis was conducted to identify demographic predictors associated with high perceived barriers to CRC screening (Table 5). The model was statistically significant (χ^2^ = 28.353, *p* = 0.005), indicating that the included variables contributed to explaining variations in screening barriers. The Nagelkerke *R*^2^ value of 0.090 suggests that the model accounted for approximately 9% of the variance in high screening barriers. Individuals who had never been screened had significantly higher odds of reporting barriers (odds ratio [OR] = 0.371, *p* = 0.005), suggesting that prior screening reduces perceived obstacles.

**Table 5:** Logistic regression analysis of predictors of high barriers to CRC screening.

Predictor	Odds Ratio (OR)	95% CI for OR	*p*-value
Gender	1.132	0.714–1.794	0.599
Education			0.463
Intermediate school	0.655	0.022–19.885	0.808
Secondary school	1.644	0.132–20.525	0.699
Undergraduate degree	2.439	0.200–29.726	0.485
Postgraduate degree	2.462	0.195–31.090	0.486
Residence	0.535	0.212–1.347	0.184
Family History of CRC	1.117	0.655–1.905	0.684
Ever Screened for CRC	0.371	0.185–0.744	0.005*
Age group			0.081*
18–30 (Reference)	1.00	–	–
31–40	0.481	0.233–0.993	0.048*
41–50	0.473	0.231–0.966	0.040*
51–60	0.434	0.204–0.926	0.031*
More than 60	0.302	0.131–0.697	0.005*

*p < 0.05 indicates statistical significance (marked with *).

Age was also a significant predictor of perceived barriers (*p* = 0.081). Compared to individuals aged 18–30 years, participants in older age groups had progressively lower odds of perceiving high barriers (ORs ranging from 0.302 to 0.481, *p* < 0.05). This trend indicates that younger individuals were more likely to report difficulties in accessing or undergoing CRC screening.

Other demographic factors, including gender, education level, and place of residence, were not significantly associated with perceived barriers. Males and females reported similar levels of screening barriers (OR = 1.132, *p* = 0.599), and education level did not significantly influence the likelihood of experiencing high barriers (*p* = 0.463). Additionally, no significant association was found between residence in core versus peripheral areas and perceived barriers (OR = 0.535, *p* = 0.184). Similarly, having a family history of CRC did not significantly affect perceived screening obstacles (OR = 1.117, *p* = 0.684).

The Hosmer–Lemeshow goodness-of-fit test was non-significant (*p* = 0.872), suggesting an adequate model fit. The overall classification accuracy of the model was 65.3%, with higher accuracy in predicting high barriers (91.0%) compared to low barriers (27.5%).

## 4 Discussion

### 4.1 Summary of findings

This study aimed to determine the barriers to colorectal cancer screening among adults in the Eastern Province of Saudi Arabia. The proportion of respondents reporting a positive family history of colorectal cancer in Saudi Arabia varies between approximately 12.8 and 20.4% across different population groups (9, 14). Our observed rate of 20% supports the representativeness of our sample despite the use of convenience sampling.

Perceived barriers clustered into three main domains—Personal Fears, Lack of Knowledge, and Healthcare Barriers—which together explained most of the variance in participants’ responses. The internal consistency of the first two domains was moderate to good, while the healthcare barriers domain showed greater variability, suggesting diverse participant experiences.

Our factor analysis identified “personal fear” as a distinct and prominent barrier domain encompassing concerns such as fear of diagnosis, anxiety about screening procedures, and embarrassment. This factor accounted for 34.8% of the total variance, highlighting its prominent role as a barrier to colorectal cancer screening. This finding aligns closely with previous research highlighting psychological barriers as critical impediments to screening uptake. For instance, Honein-AbouHaidar et al. (15) identified fear of cancer diagnosis and fear of invasive procedures as key deterrents, reducing participation in screening programs globally. Similarly, Galal et al. (9) reported that among Saudi adults, fear-related concerns significantly impact willingness to undergo screening, emphasizing the cultural and regional relevance of this barrier. In a US-based study, Sung et al. also demonstrated that personal fear, including embarrassment and anxiety about colonoscopy procedures, adversely affects screening adherence (16). Our results reinforce the necessity of addressing personal fears through targeted education and reassurance to improve screening uptake, particularly in the Saudi context where these concerns are pronounced.

Our study identified lack of knowledge as a significant barrier to colorectal cancer screening, explaining 23.7% of the variance in perceived obstacles. This finding is consistent with previous research. Similar to Galal et al. (9) who reported low awareness of CRC symptoms and screening modalities among Saudi adults, our participants exhibited limited understanding of CRC risks and the benefits of early detection. Honein-AbouHaidar et al. (15) emphasize that insufficient knowledge remains a primary psychological barrier internationally, impeding screening uptake. Furthermore, Wee et al. (17) found that knowledge gaps about CRC risk factors and screening tests contribute substantially to low participation rates, aligning with our findings. Collectively, these studies and our analysis underscore the critical need for targeted educational interventions to improve awareness and ultimately enhance CRC screening adherence in Saudi Arabia.

Healthcare system barriers accounted for 19.1% of the reported obstacles to colorectal cancer screening in our study, representing an important though comparatively smaller contributor to the overall variance. This finding aligns with previous research demonstrating that healthcare-related issues—such as inadequate physician recommendation, limited access to screening facilities, and logistical challenges—play a critical role in deterring individuals from participating in CRC screening programs. For instance, Galal et al. (9) identified healthcare system barriers as key contributors to low screening rates among older Saudi adults, emphasizing the need for improved healthcare provider engagement and service accessibility. Similarly, Honein-AbouHaidar et al. (15) found that limited provider recommendation and organizational challenges significantly affected screening behaviors in Canadian populations. These studies collectively reinforce our findings that addressing healthcare system barriers is essential for enhancing CRC screening uptake and improving early detection outcomes.

### 4.2 Implications

The current colorectal cancer screening guidelines in Saudi Arabia recommend a two-stage approach for average-risk individuals aged 45 to 75 years, beginning with an annual fecal immunochemical test (FIT) (18). Individuals with a positive FIT result are referred for diagnostic colonoscopy to confirm diagnosis and guide further management (18). High-risk individuals, including those with family history or other risk factors, are prioritized for direct colonoscopy screening (18).

The findings of this study align with the previous studies on barriers to CRC screening in Saudi Arabia and worldwide. The most frequent reported barriers in this study were the absence of symptoms, ignorance, and fear-related problems, which is consistent with the findings of similar studies. For instance, a study in Riyadh established that non-adherence was mainly due to the belief that screening was only necessary for symptomatic people, which led to delayed diagnoses and underlined the need for education about the importance of early detection (2).

In the same manner, lack of knowledge on the part of the community regarding CRC and its screening was noted as one of the major barriers in this study, as it has been in national studies (4, 8, 19). Nevertheless, the current efforts have not been effective in ensuring that all the members of the population are reached, and so community-based interventions may be required (1, 20).

Psychological barriers, especially fear of results and painful procedures, were most often reported to be present in both local and international studies (9, 10, 19). Avoidance due to fear is well-documented in cancer screening studies, particularly for invasive procedures such as colonoscopy (21). Patient education, accounts of people who have gone through the process, and words of comfort from the doctor may help calm the patient’s nerves and increase the chance of participation.

Education level was significantly associated with screening barriers, and lower educated participants reported more absence of knowledge, more difficulties in scheduling and transportation, and lack of confidence in healthcare providers. These findings are consistent with the literature, which shows that lower educational level is associated with lower knowledge and susceptibility to misinformation in healthcare (3, 8, 11). The physician’s recommendation and perceived lack of knowledge among providers were also found to be significant barriers, which is consistent with previous research that has established that provider endorsement is an important determinant of screening compliance (4, 22). Improving physician–patient communication and integrating CRC screening discussions into routine care could enhance adherence.

Unlike some studies that identified financial constraints as a primary barrier, our study found them less frequently reported (9, 23). This could be because of the government-funded healthcare services in Saudi Arabia where most people eligible for the screening pay little or nothing at all. Some participants did, however, have financial concerns, especially the young participants, which indicates that hidden costs such as transportation and time off work may also act as a deterrent. These concerns reflect broader healthcare system barriers related to access and affordability. Younger participants (≤40 years) more frequently cited knowledge-based barriers (46.2% vs. 32.7%) and financial constraints (21.5% vs. 10.7%), including concerns over transportation and time off work. The misconception that screening is unnecessary without symptoms was also more prevalent in this group (15.8% vs. 7.1%), highlighting the need for early education and employer-supported screening programs (4, 19).

Thus, psychological support and education can be offered to help overcome the screening hesitancy, especially in those with fear-related concerns. The strong relationship between awareness and reluctance to be screened suggests that public health campaigns and provider education are needed. Improving patient experience and screening access, as well as addressing financial and logistical barriers, may also improve participation rates.

Perceived barriers to CRC screening varied by gender, age, and education level and clustered into the domains of Personal Fears, Lack of Knowledge, and Healthcare System Barriers. Within Personal Fears, women were more likely than men to report fear of results (32.5% vs. 24.8%), painful procedures (27.9% vs. 19.1%), and embarrassment (31.8% vs. 22.4%), emphasizing the need for reassurance strategies such as counseling and patient testimonials (8, 9). Fear of results (28.9%) and painful procedures (20.6%) represent key psychological barriers within this domain that can be managed by patient education, decision aids, and patient enablement (8, 19). Advertisements that encourage people to be tested for CRC using non-invasive methods such as the FIT may also be helpful in reducing the fear of colonoscopy (10). In the Lack of Knowledge domain, lower education levels were associated with greater lack of awareness (48.9% vs. 22.4%) and concerns about healthcare provider knowledge (19.7% vs. 10.4%) (10, 22). Strengthening physician–patient communication and community-based education may bridge these gaps. Tailored interventions addressing psychological concerns in women, reinforcing early screening among younger individuals, and improving awareness in lower-education groups could significantly increase CRC screening rates.

Our logistic regression analysis revealed that participants who had previously undergone CRC screening were significantly less likely to report high barriers, underscoring the role of screening experience in reducing perceived obstacles. Since the current guidelines recommend annual fecal immunochemical testing (FIT), even a single experience with this less invasive screening method may help alleviate fears and misconceptions, thereby lowering perceived barriers and potentially increasing future screening uptake. Additionally, older age groups showed lower odds of high barriers, indicating that younger adults may face more challenges to screening participation. These findings highlight the importance of encouraging initial FIT screening and developing targeted interventions for younger populations to improve overall adherence to CRC screening programs.

The findings also reveal important potential targets for public health initiatives to address low rates of CRC screening. These findings indicate that knowledge-related factors, such as unawareness of symptoms of CRC (39.1%) and low levels of public awareness campaigns (35.7%), are the main challenges. Thus, mass awareness campaigns through social media, community health campaigns, and primary care services are recommended to raise awareness about the issue (2, 4).

### 4.3 Strengths and limitations

While these findings provide valuable insights, it is important to consider the strengths and limitations of this study. This study is significant in offering important information on the barriers to CRC screening in Saudi Arabia by using a large sample (*N* = 412) and a previously utilized survey instrument to ensure data reliability. The use of exploratory factor analysis (EFA) revealed distinct screening barriers such as psychological, knowledge-based, and healthcare-related barriers, which provided a richer understanding of the results.

This study addresses key research gaps identified in the introduction by providing a detailed analysis of colorectal cancer screening barriers with particular attention to demographic variation and rigorous barrier categorization. While prior research often lacked in-depth demographic stratification or relied on broad barrier assessments, our findings reveal significant demographic influences on perceived barriers. Specifically, personal fears were more pronounced among women, younger adults reported greater knowledge- and finance-related barriers, and lower educational attainment correlated with both knowledge deficits and healthcare access issues.

Moreover, the use of exploratory factor analysis with PCA-Varimax rotation enabled the empirical identification of three distinct and meaningful barrier domains—Personal Fears, Lack of Knowledge, and Healthcare Barriers. This multidimensional approach advances beyond examining individual barriers in isolation, facilitating a clearer understanding of the complex interplay of factors affecting screening behaviors. By combining statistical methodology with nuanced demographic insights, this study contributes a comprehensive framework to inform tailored, effective public health interventions for colorectal cancer screening in the Saudi context.

The study has some limitations, however. The use of self-reported data may have led to recall and social desirability bias. Furthermore, the convenience sampling strategy may have failed to capture some sections of the population, for instance, the older people who may not have easy access to digital facilities. In addition, the cross-sectional design does not allow for causal relationships to be established, as barriers are assessed at one point in time. Also, our sample size calculation was based on the overall population and did not specifically account for subgroup analyses. Consequently, some subgroup comparisons may be underpowered. Future studies with larger sample sizes are needed to confirm these subgroup-specific findings. Moreover, the relatively low internal consistency of the healthcare-related barriers subscale (*α* = 0.522) suggests that the items may not reflect a single underlying construct but instead capture a range of distinct challenges faced by participants. These may include previous negative healthcare experiences, difficulty scheduling appointments, or cost-related concerns that do not necessarily co-occur. A similar finding was reported by Imran et al. (8) in a Saudi cohort, where the same alpha value was observed. This suggests that such healthcare barriers are complex and context-dependent, and future studies may benefit from separating structural from experiential subdomains or refining the item pool to improve internal coherence.

### 4.4 Recommendations

Based on these findings and limitations, the following recommendations are proposed to improve CRC screening rates in Saudi Arabia. Longitudinal research should be conducted to examine the dynamics of screening barriers, and qualitative research should be employed to gain in-depth understanding of individuals’ experiences to develop specific intervention strategies.

From a policy perspective, increasing the effectiveness of physician-driven screening recommendations is important, as provider engagement is a key predictor of adherence (4, 22). Routine CRC discussions in primary care visits can normalize screening behaviors. Additionally, expanding access to screening, especially in underserved areas, is vital (9, 21).

To improve screening uptake, particularly among never-screened individuals, we recommend expanding workplace and community-based screening initiatives. These programs should be implemented in collaboration with primary healthcare centers and digital health platforms to support outreach, education, and follow-up. Tailored awareness campaigns addressing local cultural and educational barriers are also essential, especially given the persistently low screening rates for colorectal cancer in Saudi Arabia.

To align with current Saudi CRC screening guidelines recommending annual fecal immunochemical testing (FIT) as the initial screening modality, we emphasize promoting FIT uptake to reduce psychological barriers such as fear, embarrassment, and procedural anxiety. FIT is less invasive, more convenient, and requires less time than colonoscopy, helping address key personal fears identified in our study. Furthermore, increasing FIT accessibility and streamlining follow-up colonoscopy pathways can mitigate healthcare system barriers, enhancing overall screening adherence. Integrating FIT promotion with tailored education and improved healthcare provider engagement represents a practical strategy to improve CRC screening rates in Saudi Arabia.

These actions align with the national Health Sector Transformation Program (2021–2025), part of Saudi Arabia’s Vision 2030 framework, which prioritizes prevention, improved access, and community engagement in healthcare reform (24).

## 5 Conclusion

This study highlights key public health challenges related to CRC screening uptake in Saudi Arabia, particularly psychological barriers, limited knowledge, and healthcare access issues. These findings underscore the need for targeted, system-level interventions to improve early detection and reduce preventable mortality. Populations with lower education levels are particularly vulnerable, often due to misconceptions about screening and lack of provider engagement.

Based on these findings, we recommend integrating CRC screening into routine primary care visits through family physicians, especially in public healthcare settings. Expanding the use of non-invasive screening tools such as fecal immunochemical tests (FIT) could improve uptake among hesitant individuals. Additionally, targeted public awareness campaigns should focus on younger adults, less-educated groups, and workplace-based outreach to close participation gaps. These steps are critical for transitioning from opportunistic to organized national screening programs, ultimately improving early diagnosis rates and long-term outcomes.

## Data Availability

The raw data supporting the conclusions of this article will be made available by the authors, without undue reservation.
